# Organic‐Inorganic Perovskite Films and Efficient Planar Heterojunction Solar Cells by Magnetron Sputtering

**DOI:** 10.1002/advs.202102081

**Published:** 2021-09-16

**Authors:** Bo Gao, Jing Hu, Sheng Tang, Xinyu Xiao, Hunglin Chen, Zhuang Zuo, Qi Qi, Zongyang Peng, Jianchun Wen, Dechun Zou

**Affiliations:** ^1^ Beijing National Laboratory for Molecular Sciences Key Laboratory of Polymer Chemistry and Physics of Ministry of Education Center for Soft Matter Science and Engineering College of Chemistry and Molecular Engineering Peking University Beijing 100871 China; ^2^ Beijing Engineering Research Center for Active Matrix Display Peking University Beijing 100871 China

**Keywords:** magnetron sputtering, perovskite films, solar cells, solution‐free method

## Abstract

Organic–inorganic halide perovskites have been widely used in photovoltaic technologies. Despite tremendous progress in their efficiency and stability, perovskite solar cells (PSCs) are still facing the challenges of upscaling and stability for practical applications. As a mature film preparation technology, magnetron sputtering has been widely used to prepare metals, metallic oxides, and some semiconductor films, which has great application potential in the fabrication of PSCs. Here, a unique technology where high‐quality perovskite films are prepared via magnetron sputtering for controllable composition, solvent‐free, large‐area, and massive production, is presented. This strategy transforms the perovskite materials from powder to thin films by magnetron sputtering and post‐treatment (vapor‐assisted treatment with methanaminium iodide gas and methylamine gas treatment), which is greatly favorable to manufacture tandem solar cells. The power conversion efficiency (PCE) of PSCs with perovskite films fabricated by magnetron sputtering is 6.14%. After optimization, high‐performance perovskite films with excellent electronic properties are obtained and stable PSCs with excellent reproducibility are realized, showing a PCE of up to 15.22%. The entirely novel synthetic approach opens up a new and promising way to achieve high‐throughput magnetron sputtering for large‐area production in commercial applications of planar heterojunction and tandem PSCs.

## Graphical abstract

The mechanosynthesized perovskite powder is used to fabricate films and solar cells by magnetron sputtering. The stable PSCs with excellent reproducibility show a PCE of up to 15.22%. This approach realizes solvent‐free preparation of perovskite films with controllable composition, which would open up a promising way to achieve high‐throughput magnetron sputtering for large‐area production of planar heterojunction and tandem PSCs.

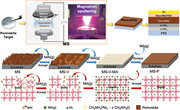

## 1 Introduction

Metal halide perovskites (*ABX*_3_, where *A* is an organic cation, *B* is a metal cation, and *X* is a halide ion) with high absorption coefficients^[^ ^1^ ^]^ and superb charge carrier mobility^[^ ^2^ ^]^ have been widely used in photovoltaic technologies. The efficiency of perovskite solar cells (PSCs) has risen from 3.8% to 25.5%,^[^ ^3^, ^4^ ^]^ approaching the high value of silicon‐based solar cells. The preparation of high‐quality perovskite thin films, reducing charge‐recombination loss inside the perovskite layer and at the layer contacts, plays a significant role on the performance of the resulting solar cells, which have attracted great attention in recent years.^[^ ^5^, ^6^, ^7^, ^8^, ^9^ ^]^ The solvent‐based process with one‐ or two‐step spin coating is the most common deposition method to form perovskite thin films due to its efficiency, relative low cost, and simple process.^[^ ^10^, ^11^, ^12^ ^]^ Jeon et al.^[^ ^13^ ^]^ reported that solvent engineering through the one‐step method for extremely uniform and dense perovskite layers with toluene drop‐casting led to remarkably improved PSCs. Other strategies, such as diverse perovskite precursors,^[^ ^14^, ^15^ ^]^ antisolvent treatment,^[^ ^16^, ^17^ ^]^ solvent annealing,^[^ ^18^ ^]^ and additive engineering,^[^ ^19^, ^20^ ^]^ have been applied to improve the quality of perovskite thin films. A sequential deposition method for high‐performance perovskite‐sensitized solar cells to permit much improved control over the formation of perovskite films was first reported by Burschka et al.^[^ ^21^ ^]^ Two‐step deposition methods have also enjoyed widespread success in the improvement of perovskite thin films for high‐performance PSCs.^[^ ^22^, ^23^, ^24^ ^]^ Scalable coating methods, such as spray‐coating,^[^ ^25^ ^]^ blade‐coating,^[^ ^26^ ^]^ slot‐die coating,^[^ ^27^ ^]^ and soft‐cover deposition,^[^ ^28^ ^]^ were proposed to fabricate high‐performance PSCs. Snaith et al.^[^ ^29^ ^]^ first demonstrated a solution‐free method to fabricate perovskite films for efficient PSCs through vapor deposition, wherein perovskite precursors were thermally evaporated in a vacuum chamber and deposited on a substrate. Jiang et al.^[^ ^30^ ^]^ reported mixed cation PSCs by stack‐sequence chemical vapor deposition with self‐passivation and gradient absorption layer. Vapor‐based processes are a promising option for deposition of metal halide PSCs in industrial environments, as they are able to deposit large area and uniform layers without the need for the use of (potentially toxic) solvents, which prevent the evolution of gain boundaries, surface defects, and groups in these materials.^[^ ^31^, ^32^ ^]^ These researches have led to unprecedented progress in PSCs with excellent performance on the basis of an organic–inorganic hybrid lead halide perovskite layer.

The next big question is whether high‐efficiency PSCs could be transformed from lab preparation to commercial scale high‐throughput production with minimal efficiency loss. Although there have been researches on the preparation of perovskite films, the solution‐free, long‐term continuous, and stable deposition cannot be achieved. In fact, the current industrial production of OLEDs and other devices has encountered such a problem, and the quality stability of coating films between different batches is a great challenge. In addition, despite the vast progresses that has been achieved on the film formation of high‐performance PSCs, the facile preparation of high‐quality and large‐area perovskite thin films with savings on raw materials, no toxic solvents, and simple equipment remains the biggest challenge in commercial and industrial application.^[^ ^33^, ^34^, ^35^ ^]^ The facial formation of lead halide perovskite thin films and their device architectures should be further deeply explored to make PSCs a relevant alternative technology in practical applications. In the field of thin film preparation, magnetron sputtering, as a mature film preparation technology, has been widely used to prepare metals, metallic oxides, and some semiconductor films for commercial and industrial applications.^[^ ^36^, ^37^ ^]^ The magnetron sputtering technique is capable of massive large‐area production compatible with current commercial and industrial applications, with low cost, high utilization ratio of the object, solvent‐free preparation, and easy controllability.^[^ ^38^ ^]^ In the present, magnetron sputtering is mainly used to prepare other active layers (electron transport layer, hole transport layer, etc.) of solar cells, rather than the perovskite thin films with excellent photoelectric properties. If the perovskite films can be prepared by magnetron sputtering for high‐efficiency solar cells, the industrial application of PSCs will be greatly promoted.

Herein, a regular planar structure of PSCs with a solution‐free preparation of perovskite active layers was developed through magnetron sputtering. This strategy transformed the perovskite materials from powder to thin film by magnetron sputtering for high‐performance solar cells. Perovskite thin films, which are extremely uniform and dense in multiple devices, could be prepared using this method in a few minutes. The CH_3_NH_3_PbI_3_ perovskite powder was first used to prepare perovskite thin films and the devices were further fabricated. The preparation conditions of perovskite films by magnetron sputtering technology and the post‐treatment process were explored to enhance the power conversion efficiencies (PCEs) up to 15%. This enhancement led to an entirely novel synthetic approach to the preparation of perovskite films with controllable composition, solvent‐free preparation, and massive large‐area production in commercial and industrial applications.

## 2 Fabrication of Perovskite Thin Films and PSCs

**Figure 1:**
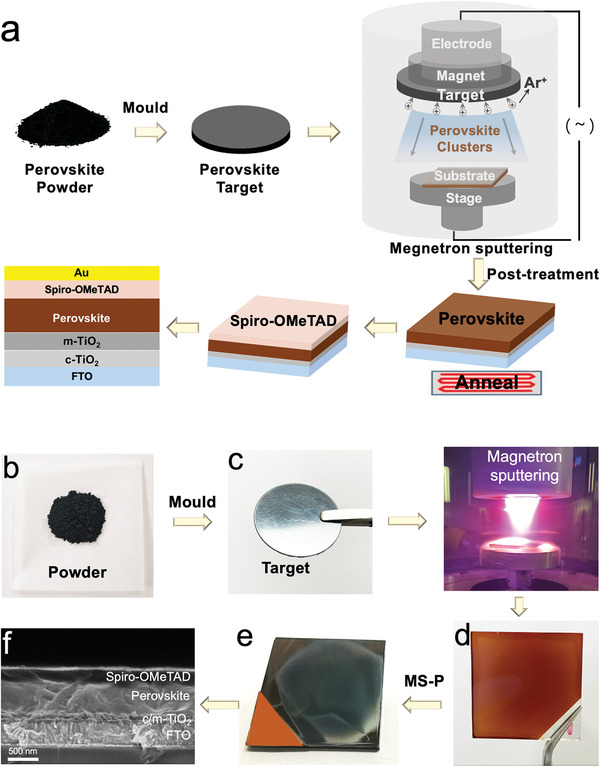
a) The scheme of the perovskite layer preparation process via sputtering and the post‐treated process. b) Perovskite powder. c) Perovskite Target. d) Perovskite thin film by magnetron sputtering. e) Perovskite thin film by magnetron sputtering and post‐treatment. f) Cross‐sectional SEM image of PSCs. The thicknesses of compact TiO_2_ (c‐TiO_2_), mesoporous TiO_2_ (m‐TiO_2_), perovskite thin film, spiro‐OMeTAD, and Au electrode are ≈50, ≈100, ≈400, ≈200, and ≈60 nm, respectively.

PSCs were fabricated on a fluorine‐doped tin dioxide (FTO)‐coated glass with perovskite (CH_3_NH_3_PbI_3_, MAPbI_3_) thin films efficiently prepared by magnetron sputtering to increase their commercial and industrial applications. The magnetron sputtering technology was applied on the preparation of perovskite films to form high‐quality active layers for high‐performance PSCs. The process is illustrated in **Figure** **1**a. Perovskite materials could be efficiently prepared via mechanosynthesis method.^[^ ^39^, ^40^ ^]^ The mechanosynthesized perovskite powder was first pressed into a specific shape of target by molding. The perovskite target was installed on a magnetron sputtering instrument and then sputtered on the substrate to fabricate perovskite thin films. During the sputtering process, Ar^+^ ions bombarded the target material to form perovskite clusters, which were deposited on the substrate to form thin films (MSMAPbI_3_) (Figure 1a,d). After the post‐treatment (MSMAPbI_3_‐P), a high‐quality film was formed on the substrate. The post‐treatment mainly included vapor‐assisted treatment with methanaminium iodide (MAI) gas (MSMAPbI_3_‐V) and further repair of film defects with methylamine (MA) gas treatment, as shown in Figure S1, Supporting Information. The formation mechanism of perovskite thin films with methylamine (MA) gas treatment is shown in Figure S2, Supporting Information. The process of film preparation exhibited the characteristics of solvent‐free, simple, and efficient preparation of large‐area perovskite films. The fabrication of PSC devices included spin coating of TiO_2_ and spiro‐OMeTAD and magnetron sputtering of Au.

Experimental results indicated that excessive applied voltage led to sputter particles with a large size, thus resulting in rough films, whilst too low voltage led to slow sputter, thus resulting in perovskite decomposition. Besides, the thickness of perovskite film is critical for device performance and it could be easily controlled by the sputtering time. The perovskite film thickness was measured as the time of sputtering methylammonium lead halide (MAPbI_3_) prepared via mechanosynthesis on FTO/TiO_2_ substrates, as shown in Table S1, Supporting Information. Therefore, the optimum conditions to form ≈400–500 nm of perovskite layer thickness were as follows: applied voltage of 800 V, pressure of 9.6 Pa, and 5 min of time. These procedures were all carried out under a humidity of below 30% or in nitrogen atmosphere.

Optical photos of the transformation from perovskite powder to thin film are shown in Figure 1b–e. The perovskite powders were also characterized, and the results are shown in Figure S3, Supporting Information. As shown in Figure 1b,c, mechanosynthesized perovskite powder could be easily pressed into a specific shape of target with metallic luster by molding. The solid perovskite was then converted into thin films by magnetron sputtering. After post‐treatment, the macroscopic morphology changed significantly; the color of perovskite thin films was obviously darkened (Figure 1d,e). The changes after post‐treatment indicated that post‐processing could form high‐quality perovskite films.

Figure 1f presents a cross‐sectional scanning electron microscopy (SEM) image of a PSC. The PSC consists of FTO, compact titanium dioxide (c‐TiO_2_), mesoporous titanium dioxide (m‐TiO_2_), perovskite thin film, spiro‐OMeTAD layers, and Au electrode. A smooth and uniform perovskite layer with a thickness of ∼400 nm prepared via magnetron sputtering technology on the top of TiO_2_ layers could be clearly observed.

### 2.1 Structure and Properties of Perovskite Thin Films

**Figure 2:**
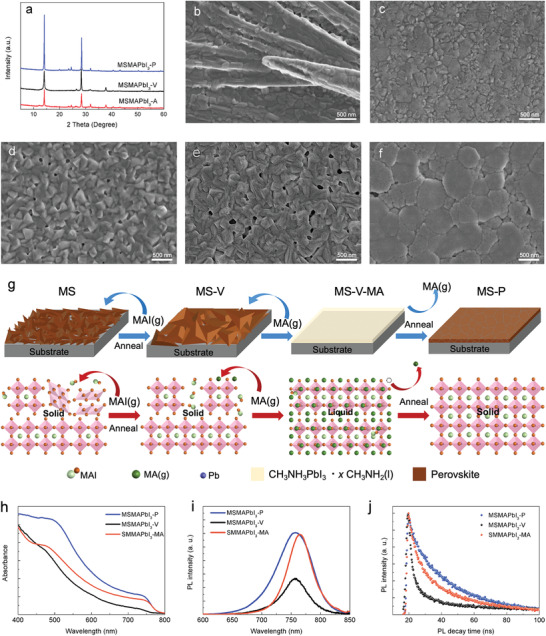
Microstructure and property characterizations of perovskite thin films fabricated by magnetron sputtering. a) XRD patterns of MSMAPbI_3_‐A, MSMAPbI_3_‐V, and MSMAPbI_3_‐P films. Top scanning electron microscopic images: b) SMMAPbI_3_ film. c) SMMAPbI_3_‐MA film. d) MSMAPbI_3_‐A film. e) MSMAPbI_3_‐V film. f) MSMAPbI_3_‐P film. The same scale bar and label apply to all images in a column. g) Crystal growth mechanisms of perovskite thin films. h) Ultraviolet‐visible–near‐infrared (UV–vis) absorption spectra of SMMAPbI_3_‐MA, MSMAPbI_3_, and MSMAPbI_3_‐P films. i) Steady‐state photoluminescence (PL) spectra of SMMAPbI_3_‐MA, MSMAPbI_3_‐V, and MSMAPbI_3_‐P films. j) Time‐resolved PL decays of SMMAPbI_3_‐MA, MSMAPbI_3_‐V, and MSMAPbI_3_‐P films.

X‐ray diffraction (XRD) is one of the most powerful tools for the characterization of the perovskite structure. It was carried out (details shown in the Experimental Section) in this study to gain insights into the component of thin films fabricated via magnetron sputtering. **Figure** **2**a shows the diffractograms of the perovskite films. The post‐treatments yielded a gradual increase in the diffraction peak intensity. As shown in Figure 2a, in XRD spectrum of perovskite thin film fabricated by magnetron sputtering and annealing (MSMAPbI_3_‐A), the specific diffraction peak of PbI_2_ still existed. The excess PbI_2_ was believed to be the sublimation of MAI in the process of annealing. The specific diffraction peak of PbI_2_ disappeared in XRD spectrum of perovskite thin film fabricated by magnetron sputtering and vapor‐assisted treatment, which indicated the excess PbI_2_ was reacted with MAI gas. Two stronger diffraction peaks were found in the XRD pattern of MSMAPbI_3_‐P film, indicating that post‐treatment could significantly improve the quality of the films. The results were further proven by SEM characterization, as shown in Figure 2. Perovskite thin films prepared via solution method (SMMAPbI_3_) were chosen as the control. Figure 2b,c shows their surface properties. After the film formation of MAPbI_3_ by spinning coating and annealing, the crystal structure of the perovskite was in a needle‐like arrangement with low coverage (Figure 2b). MA gas treatment (SMMAPbI_3_‐MA) could improve the morphology of the films, with high coverage and smoothness in Figure 2c. Meanwhile, MSMAPbI_3_‐P film (Figure 2f) exhibited continuous and compact perovskite thin films with large grain size and less defects compared with MSMAPbI_3_‐A (Figure 2d) and MSMAPbI_3_‐V (Figure 2e) films because post‐treatment could repair the film. The fabrication steps for crystal growth mechanisms of perovskite thin films were also shown in Figure 2g. As shown in Figure 2a,e,g, the step of vapor‐assisted treatment can ensure that the film is composed of MAPbI_3_ perovskite with pure phase. Therefore, post‐treatment is not only necessary but also in line with the original idea of solvent‐free preparation of perovskite films. Especially, MA gas treatment is the key to prepare high quality perovskite films, which can transfer the film from individual particles into a continuous entirety, in Figure 2d,f,g. This process is related to Equations (1) and (2):^[^ ^41^ ^]^

(1) $$\begin{array}{c} CH_{3}NH_{3}\mathrm{Pb}I_{3}\left(s\right)+xCH_{3}NH_{2}\left(g\right)⟶CH_{3}NH_{3}\mathrm{Pb}I_{3}\cdot xCH_{3}NH_{2}\left(l\right) \end{array}$$

(2) $$\begin{array}{c} CH_{3}NH_{3}\mathrm{Pb}I_{3}\cdot xCH_{3}NH_{2}\left(l\right)⟶CH_{3}NH_{3}\mathrm{Pb}I_{3}\left(s\right)+xCH_{3}NH_{2}\left(g\right) \end{array}$$

The lower multiple SEM images shown in Figure S4, Supporting Information, further indicated that the MAPbI_3_ perovskite films prepared via solution method had poor coverage. The XRD and SEM characterizations of the films with and without post‐treatment are discussed in detail in Figure S5, Supporting Information. Comparison between Figure 2c,f showed that the properties of MSMAPbI_3_‐P thin film were superior to those of SMMAPbI_3_‐MA films, thereby facilitating the preparation of PSCs. Figure 2h compares the ultraviolet–visible (UV–vis) absorption spectra of SMMAPbI_3_‐MA, MSMAPbI_3_‐V, and MSMAPbI_3_‐P thin films over a wide absorption range of 400–800 nm. The different films displayed various excitonic absorption peaks associated with the properties of perovskite thin films. The UV–vis absorption spectra of MSMAPbI_3_‐P and SMMAPbI_3_‐MA films showed similar excitonic absorption peaks, indicating that the bandgap of MSMAPbI_3_‐P films was the same as that of SMMAPbI_3_‐MA films. Compared with SMMAPbI_3_‐MA perovskite films, MSMAPbI_3_‐P perovskite films clearly showed enhanced absorption, which is consistent with the enhancements in the resulting surface morphology of films, the resulting *J*_SC_ and the corresponding PSCs. Moreover, Figure S6, Supporting Information, shows the UV–vis absorption spectra of MSMAPbI_3_ and the UV–vis absorption, PL, and TRPL spectra of MSMAPbI_3_‐A films. These results revealed the high quality of the perovskite films prepared via magnetron sputtering with post‐treatment and this high quality is beneficial for conveniently preparing high‐performance solar cells in the industry. In addition, the spectrum of MSMAPbI_3_‐V films with different excitonic absorption peaks indicated the poor properties of the perovskite thin films, thereby revealing the essence of post‐treatment.

The charge‐carrier behavior of SMMAPbI_3_‐MA, MSMAPbI_3_‐V, and MSMAPbI_3_‐P perovskite films was investigated using photoluminescence (PL) spectra (Figure 2i,j). The SMMAPbI_3_‐MA film exhibited a PL peak at 764 nm, whilst the MSMAPbI_3_‐P film exhibited a PL peak at 758 nm. The blueshift in the PL spectra indicated that the defects of perovskite films could be reduced by magnetron sputtering with post‐treatment.^[^ ^42^ ^]^ In addition, the peak intensity increased from MSMAPbI_3_‐V to MSMAPbI_3_‐P, which revealed that MA gas could heal the perovskite thin films to enhance the photoelectric performance of films.

Time‐resolved PL decay measurements (Figure 2j) of SMMAPbI_3_‐MA, MSMAPbI_3_‐V, and MSMAPbI_3_‐P films were carried out to find the origin of the improved efficiency. The TRPL decay of perovskite films prepared via solution method and magnetron sputtering showed a bi‐exponential decay with a fast and a slow component. Previous studies indicated that the fast decay process was due to charge‐carrier trapped in the defect sites, whilst the slow decay process was attributed to the bimolecular recombination of photo‐generated free carriers.^[^ ^42^, ^43^ ^]^ The PL decay of the MSMAPbI_3_‐V film was very rapid due to the poor properties of the film. In addition, the spectra in Figure 2j revealed that the PL decay of the SMMAPbI_3_‐MA film was faster than that of the MSMAPbI_3_‐P film. This finding indicated a lower defect concentration and superior electronic properties in MSMAPbI_3_‐P‐based perovskite films, which is consistent with the higher FF in the corresponding PSCs.^[^ ^44^, ^45^ ^]^

### 2.2 Performance of Photovoltaic Devices

**Figure 3:**
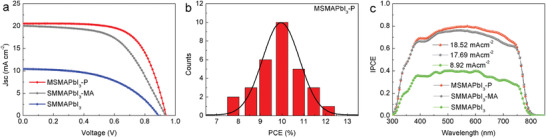
Device performance of perovskite solar cells with the structure glass/FTO/TiO_2_/perovskite/Spiro‐OMeTAD/Au. a) Device performance of MSMAPbI_3_‐P‐ and SMMAPbI_3_‐MA‐based solar cells. b) Device performance distribution for 30 devices in one batch. The red curve represents a Gaussian function fit to the data. c) Incident photon‐to‐current efficiency (IPCE) spectrum. The *J*_SC_ values of MSMAPbI_3_‐P‐, SMMAPbI_3_‐, and SMMAPbI_3_‐MA‐based devices calculated from the IPCE curve are 18.52, 17.69, and 8.92 mA cm^−2^, respectively.

The performances of PSCs obtained via magnetron sputtering and solution method are presented in **Figure** **3** and Figure S7, Supporting Information. The photovoltaic performance of MAPbI_3_‐based devices was examined. The highest PCE (measured under standard test conditions of 25 °C, simulated AM 1.5 G, and 100 mW cm^−2^) of MSMAPbI_3_‐P‐based solar cells reached 12.76%. As shown in **Table** **1** and Table S2, Supporting Information, the MSMAPbI_3_‐P‐based device showed an open‐circuit voltage (*V*_OC_) of 0.94 V, a short‐circuit current (*J*_SC_) of 20.56 mA cm^−2^, a fill factor (FF) of 0.66, and a PCE of 12.76% under backward scan. SMMAPbI_3_‐ and SMMAPbI_3_‐MA‐based solar cells were prepared as a control. The highest PCE (measured under standard test conditions of 25 °C, simulated AM 1.5 G, and 1000 Wm^−2^) of SMMAPbI_3_‐based solar cells reached 4.64% under backward scan. The device showed a *V*_OC_ of 0.89 V, a *J*_SC_ of 10.42 mA cm^−2^ and an FF of 0.50. These results were consistent with the SEM characterization. After MA gas treatment, the quality of the perovskite film was significantly enhanced, leading to an intensive increase in the performance of the resulting devices. Table 1 shows the highest PCE (10.55%, measured under backward scan) of SMMAPbI_3_‐MA‐based solar cells. The device showed *V*_OC_ of 0.94 V, *J*_SC_ of 20.05 mA cm^−2^, and FF of 0.56. These results showed that perovskite thin films prepared via magnetron sputtering could be used to fabricate high‐performance solar cells and the magnetron sputtering technology is a novel and effective method for the preparation of PSCs.

**Table 1:** Photovoltaic performance of MSMAPbI_3_‐P, SMMAPbI_3_‐MA, and SMMAPbI_3_ based solar cells

	*J*_SC_ [mA cm^−2^]	*V*_oc_ [V]	FF	PCE [%]
MSMAPbI_3_‐P	20.56	0.94	0.66	12.76
SMMAPbI_3_‐MA	20.05	0.94	0.56	10.55
SMMAPbI_3_	10.42	0.89	0.50	4.64

The PCE of one batch of MSMAPbI_3_‐P‐based devices was measured to check the reproducibility of the performance of photovoltaic devices (30 devices, Figure 3b). The PCE ranged from 8% to 13% and most of the device efficiencies were greater than 10%, indicating that the PSCs prepared via magnetron sputtering have practical application values. Incident photon‐to‐current efficiency (IPCE) was also carried out on SMMAPbI_3_‐, SMMAPbI_3_‐MA‐, and MSMAPbI_3_‐P‐based PSCs to confirm the *J*_SC_, as shown in Figure 3c. The MSMAPbI_3_‐P‐based devices exhibited a high IPCE (nearly 80%) in the visible light region. The values integrated from the IPCE characteristics of the devices closely agreed with those extracted from the *J*–*V* curves.

### 2.3 MSMAPbI_3−_ *_x_* Cl*_x_* Thin Films and Photovoltaic Performance of Devices

In an attempt to increase the performance of PSCs, Cl was introduced to improve the properties of perovskite thin films (MSMAPbI_3−_ *_x_* Cl*_x_*‐P) and the resulting devices. The fabrication of perovskite thin films and PSCs was similar to that of MSMAPbI_3_‐P in Figure 1. The effects of Cl on the properties of MSMAPbI_3−_ *_x_* Cl*_x_*‐P thin films and device performance of PSCs were investigated using various techniques.

**Figure 4:**
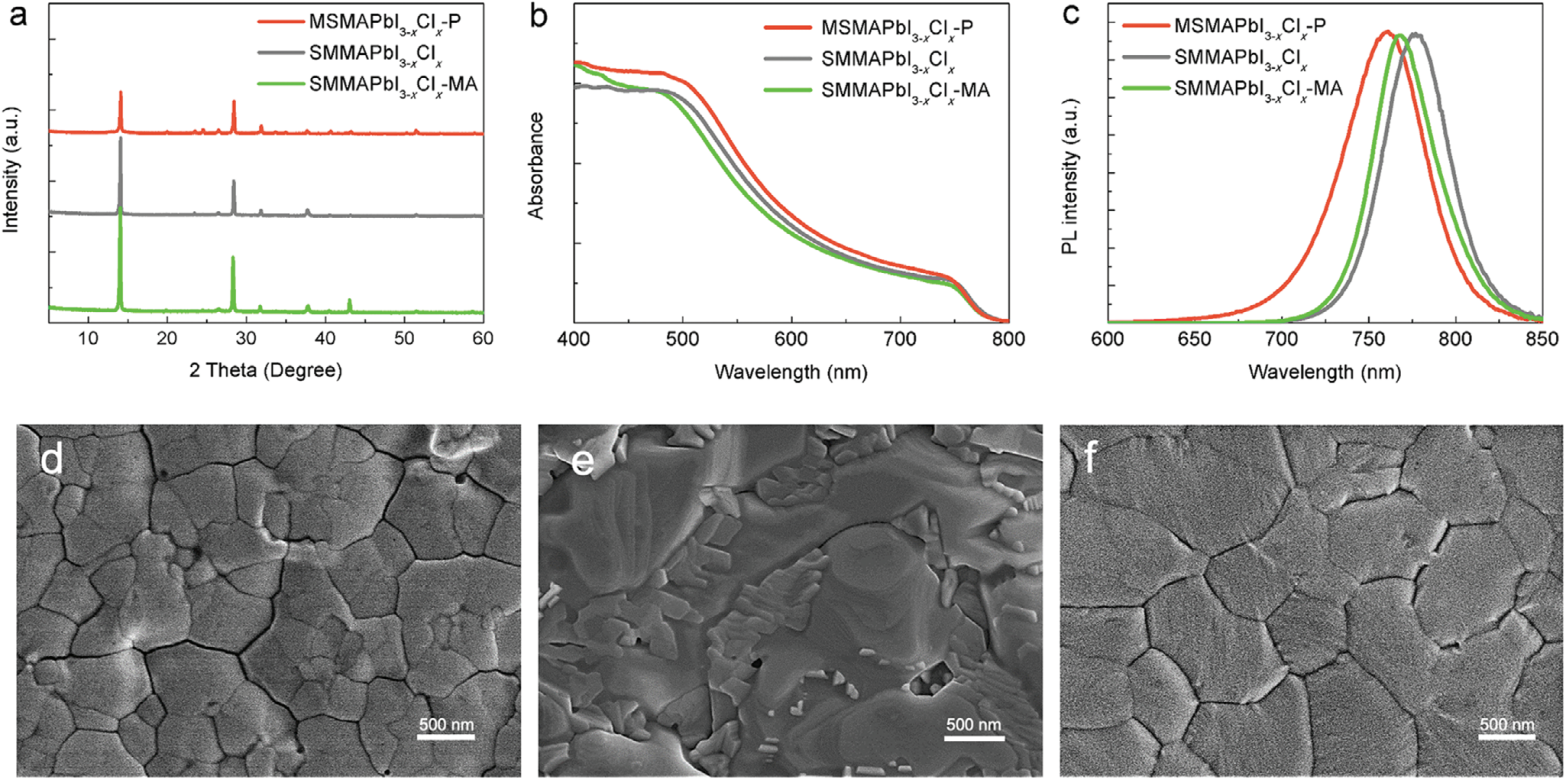
Properties of MAPbI_3−_ *_x_* Cl_x_ thin films. a) XRD patterns of perovskite film (MSMAPbI_3−_ *_x_* Cl*_x_*‐P, SMMAPbI_3−_ *_x_* Cl*_x_*, and SMMAPbI_3−_ *_x_* Cl*_x_*‐MA). b) UV–vis absorption spectra of MSMAPbI_3−_ *_x_* Cl*_x_*‐P, SMMAPbI_3−_ *_x_* Cl*_x_*, and SMMAPbI_3−_ *_x_* Cl*_x_*‐MA films. c) Steady‐state PL spectra of MSMAPbI_3−_ *_x_* Cl*_x_*‐P, SMMAPbI_3−_ *_x_* Cl*_x_*, and SMMAPbI_3−_ *_x_* Cl*_x_*‐MA films. d–f) Top scanning electron microscopic images of MSMAPbI_3−_ *_x_* Cl*_x_*‐P, SMMAPbI_3−_ *_x_* Cl*_x_*, and SMMAPbI_3−_ *_x_* Cl*_x_*‐MA films, respectively.

XRD was used to characterize the perovskite structure and the results are shown in **Figure** **4**a. The XRD pattern of SMMAPbI_3−_ *_x_* Cl*_x_*, SMMAPbI_3−_ *_x_* Cl*_x_*‐MA, and MSMAPbI_3−_ *_x_* Cl*_x_*‐P showed the same peak position at 14.11 and 28.42°. Compared with MSMAPbI_3_ (Figure 2a), the peak positions of MSMAPbI_3−_ *_x_* Cl*_x_* show increases from 14.02° to 14.11° and from 28.26° to 28.42°, indicating that Cl was successfully introduced. The UV–vis spectra in Figure 4b showed that the bandgap of the MSMAPbI_3−_ *_x_* Cl*_x_*‐P film was the same as that of the reference SMMAPbI_3−_ *_x_* Cl*_x_* and SMMAPbI_3−_ *_x_* Cl*_x_*‐MA films. Almost no further blueshift of the absorption edge was observed with the introduction of PbCl_2_ in the perovskite powder, thus revealing that a low but subsistent content of Cl was incorporated into the pristine MAPbI_3_ structure to form the hybrid MAPbI_3−_ *_x_* Cl*_x_*.^[^ ^46^ ^]^

TRPL and steady PL measurements were performed on different perovskite thin films (Figure 4c and Figure S8, Supporting Information). As shown in Figure 4c, the SMMAPbI_3−_ *_x_* Cl*_x_* and SMMAPbI_3−_ *_x_* Cl*_x_*‐MA films exhibited PL peaks at 776 and 767 nm, respectively, whilst the MSMAPbI_3−_ *_x_* Cl*_x_*‐P film exhibited a PL peak at 761 nm. The blueshift in PL spectra indicated that the perovskite films prepared by magnetron sputtering have fewer defects.^[^ ^42^ ^]^

Time‐resolved PL decay measurement of MSMAPbI_3−_ *_x_* Cl*_x_*‐P was also carried out to find the origin of the improved efficiency. The spectra revealed the PL decay of MSMAPbI_3−_ *_x_* Cl*_x_*‐P film was remarkably slower than that of MSMAPbI_3_‐P and SMMAPbI_3_‐MA films (Figure 2j) and similar to that of SMMAPbI_3−_ *_x_* Cl*_x_*‐MA film (Figure S8, Supporting Information). These results revealed a lower defect concentration and superior electronic properties in the MSMAPbI_3−_ *_x_* Cl*_x_*‐P‐based perovskite film, which could be attributed to the introduction of Cl.

SEM was performed to further investigate the influence of Cl on the perovskite thin films. As shown in Figure 4d, the surface morphologies and microstructures of MSMAPbI_3−_ *_x_* Cl*_x_*‐P changed significantly compared with those of MSMAPbI_3_‐P (Figure 2f). The obtained perovskite film showed an excellent morphology, a lacking of pinholes, and increased crystal size. The high quality of perovskite film could be attributed to the introduction of Cl during the film growth.^[^ ^47^ ^]^ In addition, the properties of the SMMAPbI_3−_ *_x_* Cl*_x_* film after MA treatment were slightly improved, leading to slightly increased PCE.

**Figure 5:**
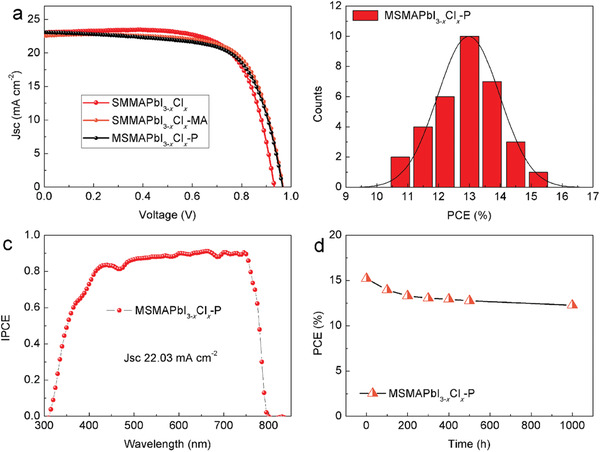
Device performance of perovskite solar cells. Device performance. a) Device performance of MSMAPbI_3−_ *_x_* Cl*_x_*‐P‐, SMMAPbI_3−_ *_x_* Cl*_x_*‐, and SMMAPbI_3−_ *_x_* Cl*_x_*‐MA‐based solar cells. b) Device performance distribution for 30 devices in one batch (MSMAPbI_3−_ *_x_* Cl*_x_*‐P‐based solar cells). The black curve represents a Gaussian function fit to the data. c) Incident photon‐to‐current efficiency (IPCE) spectrum. The *J*_SC_ values of MSMAPbI_3−_ *_x_* Cl*_x_*‐P‐based devices calculated from the IPCE curve is 22.03 mA cm^−2^. d) Evolution of PCE relative to the initial parameters for PSCs without encapsulation stored under nitrogen atmosphere and at a temperature of ≈15–25 °C.

SEM results showed an excellent morphology and lessened pinholes, which are favorable to enhance the performance of PSCs. **Figure** **5**a and Figure S9, Supporting Information, and **Table** **2** show the results of the photovoltaic performance of MSMAPbI_3−_ *_x_* Cl*_x_*‐P‐, MSMAPbI_3−_ *_x_* Cl*_x_*‐V‐ (preparation via magnetron sputtering with vapor‐assisted treatment), SMMAPbI_3−_ *_x_* Cl*_x_*‐, and SMMAPbI_3−_ *_x_* Cl*_x_*‐MA‐based devices. The photovoltaic performances of MSMAPbI_3−_ *_x_* Cl*_x_*‐P‐, SMMAPbI_3−_ *_x_* Cl*_x_*‐, and SMMAPbI_3−_ *_x_* Cl*_x_*‐MA‐based solar cells were similar. The highest PCE (measured under standard test conditions of 25 °C, simulated AM 1.5 G, and 1000 Wm^−2^) of the MSMAPbI_3−_ *_x_* Cl_x_‐P‐based device reached 15.22%, with *V*_OC_ of 0.97 V, *J*_SC_ of 23.08 mA cm^−2^, and FF of 0.68. The SMMAPbI_3−_ *_x_* Cl*_x_*‐based solar cell (*V*_OC_ of 0.93 V, a *J*_SC_ of 23.04 mA cm^−2^, and FF of 0.71) and the SMMAPbI_3−_ *_x_* Cl*_x_*‐MA‐based solar cell (*V*_OC_ of 0.97 V, *J*_SC_ of 22.53 mA cm^−2^, and FF of 0.71) also showed similar PCEs (15.21% and 15.52%, respectively). These results showed that the preparation of perovskite films via magnetron sputtering for high‐performance solar cells is comparable to the solution method and has great industrial potentials.

**Table 2:** Photovoltaic performance of MSMAPbI_3−_ *_x_* Cl*_x_*‐P, SMMAPbI_3−_ *_x_* Cl*_x_*_,_ and SMMAPbI_3−_ *_x_* Cl*_x_*‐MA based solar cells

	*J*_SC_ [mA cm^−2^]	*V*_OC_ [V]	FF	PCE [%]
MSMAPbI_3−_ *_x_* Cl*_x_*‐P	23.08	0.97	0.68	15.22
SMMAPbI_3−_ *_x_* Cl*_x_*	23.04	0.93	0.71	15.21
SMMAPbI_3−_ *_x_* Cl*_x_*‐MA	22.53	0.97	0.71	15.52

The PCE of devices of one batch was also measured to check the reproducibility of the performance of photovoltaic devices (35 devices, Figure 5b). The PCE ranged from 11% to 16% and most of the device efficiencies were greater than 13%. A series of investigations on unencapsulated devices was carried out to establish the stability of the improved MSMAPbI_3−_ *_x_* Cl*_x_*‐P material. IPCE was also performed for the MSMAPbI_3−_ *_x_* Cl*_x_*‐P‐, SMMAPbI_3−_ *_x_* Cl*_x_*‐, and SMMAPbI_3−_ *_x_* Cl*_x_*‐MA‐based devices to confirm the *J*_SC_, as shown in Figure 5c and Figure S10, Supporting Information. The IPCE of MSMAPbI_3_‐P‐based device exhibited over 85% in the visible light region. The current integrated from the IPCE characteristics of the devices closely agreed with those extracted from the *J*–*V* curves.

The long‐term stability of PSCs was investigated to evaluate the performance of MSMAPbI_3−_ *_x_* Cl*_x_*‐P‐based solar cells. The performance of the unencapsulated PSCs stored under nitrogen atmosphere and at a temperature of ≈15–25 °C for 1000 h was measured. As shown in Figure 5d, the PCE of MSMAPbI_3−_ *_x_* Cl*_x_*‐P‐based solar cells decreased from 15.27% to 12.26%, maintaining over 80% of the initial PCE after 1000 h. This finding indicated that MSMAPbI_3−_ *_x_* Cl_x_‐P‐based solar cells prepared via magnetron sputtering are relatively stable under this condition and they have more prospects of practical application.

## 3 Conclusions

In summary, a regular planar structure of PSCs with solution‐free preparation of perovskite active layers by magnetron sputtering technology was demonstrated. This technology could transform the perovskite materials from powder to thin film for high‐performance solar cells. Extremely uniform and dense perovskite thin films could be prepared using this method in a few minutes. This study not only explored the detailed conditions for film formation of MSMAPbI_3_ by magnetron sputtering but also demonstrated that introducing Cl could further improve the PCE of the resulting devices by over 15%. This improvement enabled an entirely novel synthetic approach to prepare perovskite films with controllable composition, solvent‐free preparation, and massive and large‐area production. This novel approach of preparing perovskite thin films via magnetron sputtering opened up a new and promising method for the industrial production of PSCs.

## 4 Experimental Section

### Materials

MA (CH_3_NH_2_, 40.0 wt% in ethanol) and hydroiodic acid (HI, 55.0–58.0 wt% in water, stable with 1.5 wt% hypophosphorous) and PbCl_2_ (99.9%) were purchased from Aladdin Reagent Ltd. PbI_2_ (99.0%) was purchased from Adamas Reagent Ltd. All materials were used as received without further purification.

### Methylammonium Iodide (CH_3_NH_3_I) Synthesis

Methylammonium iodide was synthesized using the method described by Lee et al.,^[^ ^48^ ^]^ where CH_3_NH_2_ (28.8 mL) and HI (20 mL) were mixed in a flask under nitrogen atmosphere in an iced bath for 2 h. The mixed solution was then evaporated at 55 °C for ≈2 h to remove the solvent. The products were recrystallized in ethanol. The crystalline powders were washed by diethyl ether three times and then dried in a vacuum oven at 60 °C overnight.

### Mechanosynthesis of MAPbI_3_ Powders and Molding the Target

MAPbI_3_ powders were synthesized by mixing the desired amount of CH_3_NH_3_I with PbI_2_ (1:1 *n*/*n*, molar ratio) in an agate jar and reacted via mechanical ball milling at room temperature (RT) under ambient conditions (speed of 400 rpm and humidity of 20–30%).^[^ ^49^ ^]^ For the introduction of Cl element, MAPbI_3_ with PbCl_2_ powders were synthesized by mixing the desired amount of CH_3_NH_3_I and PbI_2_ (1:1 *n*/*n*, molar ratio) with the mass ratios 5 wt% of PbCl_2_ in an agate jar and reacted via mechanical ball milling at room temperature (RT) under ambient conditions (speed of 400 rpm and humidity of 20–30%). The concentration of Cl could be varied by the mass of PbCl_2_, which is conveniently controlled and reproduced. The milling jars were tightly closed, inserted into the mill and the synthesis process starts with the movement of the balls. The milling balls crushed the reactants, thus providing the reaction energy by impact and friction.^[^ ^39^ ^]^

1 g of MAPbI_3_ powders or MAPbI_3_ with PbCl_2_ powders were then pressed into a specific shape of target by molding with a pressure of 60 MPa. The diameter of the perovskite target was ≈2 cm and the thickness was ≈2 mm. The surface of perovskite target was relatively smooth and the target had metallic luster.

### PSC Fabrication

An FTO glass was cleaned ultrasonically and sequentially with detergent, deionised water, acetone, and isopropanol for 30 min each. The cleaned FTO glass was treated via ultraviolet ozone for 2 min. The c‐TiO_2_ was spin coated on an FTO substrate at 2000 rpm for 45 s by using acidic ethanol solution containing tetrabutyl titanate, which was heated at 135 °C for 15 min. TiO_2_ paste (Dyesol, 18NR‐T transparent titania paste) and ethanol were mixed at a 1:6.5 mass ratio and then stirred for overnight at room temperature to obtain a TiO_2_ paste dispersion. The TiO_2_ paste dispersion was spin coated on c‐TiO_2_ at 5000 rpm for 20 s, where the pristine paste was diluted in ethanol (13.3 wt%). After drying at 135 °C for 15 min, the film was annealed at 500 °C for 30 min, providing a thickness of ≈120 nm.^[^ ^50^ ^]^ The mechanosynthesized perovskite powder (MAPbI_3_) was pressed into a specific shape of target by molding. The perovskite target was installed on a magnetron sputtering instrument and then sputtered on the substrate to fabricate perovskite thin films. After post‐treatment (MSMAPbI_3_‐P), high‐quality films were formed on the TiO_2_ layer. Spiro‐OMeTAD chlorobenzene solution was dropped on the perovskite surface and treated for 60 s by spin coating at 2000 rpm. The spiro‐OMeTAD solution was prepared by dissolving 72.3 mg of spiro‐OMeTAD into 1 mL of chlorobenzene containing 28.8 µL of 4‐tert‐butyl pyridine and 17.5 µL of lithium bis(trifluoromethanesulfonyl)imide (Li‐TFSI) solution (520 mg of Li‐TSFI in 1 mL acetonitrile, Sigma‐Aldrich, 99.8%). Finally, 80 nm of Au was formed on the spiro‐OMeTAD‐coated film by magnetron sputtering.

### Characterization of Perovskite Thin Films

The crystal structures of the perovskite thin films were characterized using an X‐ray diffractometer (D/MAX‐PC 2500, Rigaku). A Lambda 35 UV spectrometer (Perkin Elmer) was used to obtain the UV absorption spectra of the samples. The test samples were prepared on a glass. The static PL and time‐resolved PL spectra of the perovskite thin films on FTO substrates were measured using an Edinburgh FLS 980 under the irradiation of a 405 nm pulse laser.

### Device Characterization

The photovoltaic performance of the cells was measured in ambient conditions with a Keithley Model 2000 instrument under simulated AM 1.5 sunlight generated by the YSS‐5A system (Yamashita Denso, Japan). The light intensity (≈100 mW cm^−2^) of the system was calibrated with a standard silicon solar cell. IPCE measurements were performed with a solar cell monochromatic incident photon‐to‐electron conversion efficiency measurement system (SCS10‐X150‐DSSC, Zolix); the active area of the test samples was 6 mm^2^. Film morphology of the perovskite and cross‐sectional morphology of the resulting solar cells were observed using a field‐emission scanning electron microscope (S‐4800, Hitachi, Japan).

## Conflict of Interest

The authors declare no conflict of interest.

## Supporting information

Supporting Information

## Data Availability

Research data are not shared.
