# Supplementary material for: Organic‐Inorganic Perovskite Films and Efficient Planar Heterojunction Solar Cells by Magnetron Sputtering
Source: Adv Sci (Weinh). 2021 Sep 16;8(22):2102081. doi: 10.1002/advs.202102081 (PMC8596124; doi:10.1002/advs.202102081)
Supplement: Supplementary file 1 — Supporting Information [file ADVS-8-2102081-s001.pdf]

© 2021 Wiley-VCH GmbH

ADVANCED SCIENCE
Open Access

Supporting Information

for *Adv. Sci.,* DOI: 10.1002/advs.202102081

Organic-inorganic Perovskite Films and Efficient Planar Heterojunction Solar Cells by Magnetron Sputtering

*Bo Gao, Jing Hu, Sheng Tang, Xinyu Xiao, Hunglin Chen, Zhuang Zuo, Qi Qi, Zongyang Peng, Jianchun Wen, and Dechun Zou**

Organic-inorganic Perovskite Films and Efficient Planar Heterojunction Solar Cells by Magnetron Sputtering

*Bo Gao†, Jing Hu†, Sheng Tang, Xinyu Xiao, Hunglin Chen, Zhuang Zuo, Qi Qi, Zongyang Peng, Jianchun Wen, and Dechun Zou**

Beijing National Laboratory for Molecular Sciences
Key Laboratory of Polymer Chemistry and Physics of Ministry of Education
Center for Soft Matter Science and Engineering
College of Chemistry and Molecular Engineering
Peking University
Beijing 100871, China
E-mail: dczou@pku.edu.cn
Prof. D. Zou
Beijing Engineering Research Center for Active Matrix Display
Peking University
Beijing 100871, China

† These authors contributed equally to this work.

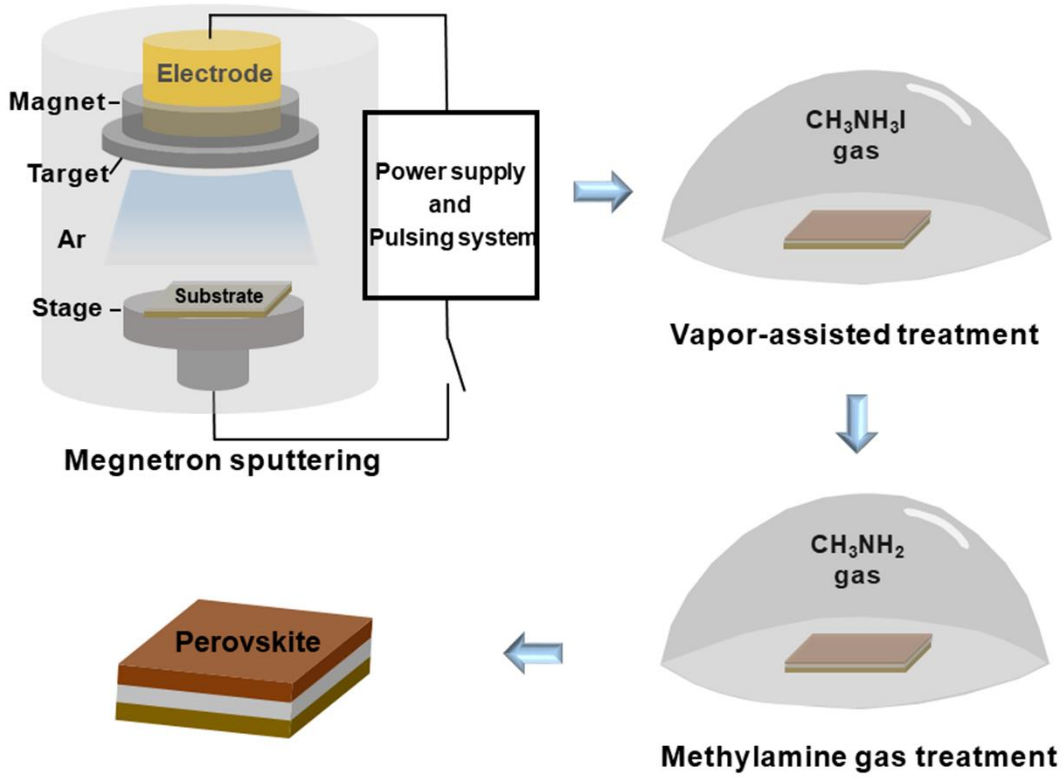

Figure S1. The process of post-treatment mainly including vapor-assisted treatment with iodomethylamine gas ($MSMAPbI_3$-V) and further repair of film defects with methylamine gas treatment ($MSMAPbI_3$-V-MA).

Chen et al.[1] demonstrated a low-temperature vapor-assisted solution process to construct polycrystalline perovskite thin films with full surface coverage, small surface roughness and grain size up to microscale. Pang et al.[2] discovered that methylamine (CH_3NH_2) could induce defect healing of $CH_3NH_2NH_3PbI_3$ perovskite thin films on the basis of ultrafast, reversible chemical reaction with CH_3NH_2 gas at room temperature,.in **Figure S2** Therefore, vapor-assisted treatment with iodomethylamine gas and methylamine gas treatment were used in the present study to prepare high-quality perovskite thin films. The preparation process of post-treatment is shown in **Figure S1.**

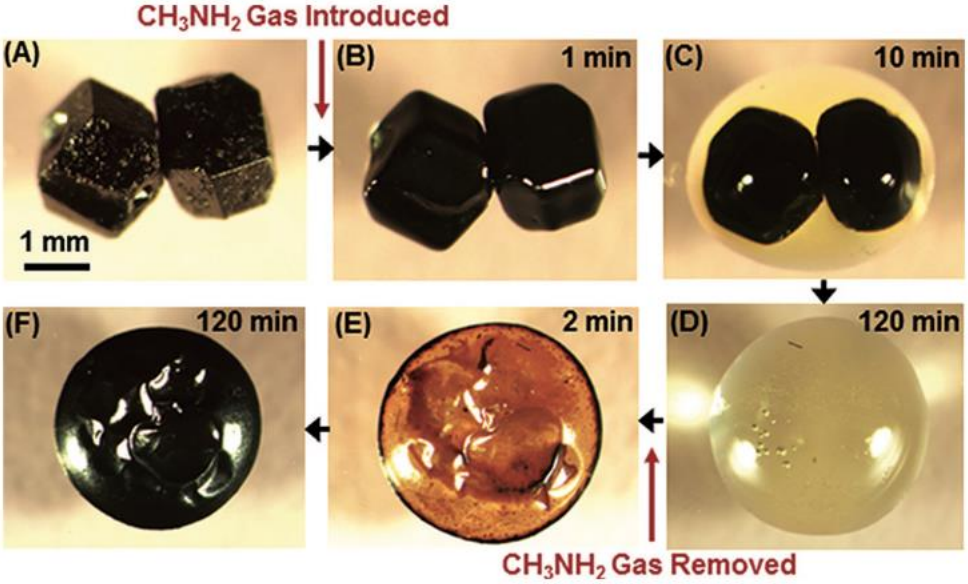

Figure S2. In situ optical microscopy of the morphology evolution of two touching $MAPbI_3$ perovskite crystals (same magnification) upon exposure to CH_3NH_2 gas and CH_3NH_2 degassing: A) before CH_3NH_2 gas exposure, B) CH_3NH_2 gas introduced, C) partial collapse of perovskite structure and conversion to liquid, D) full conversion to liquid, E) CH_3NH_2 degassing, and F) perovskite back-conversion completed[2].

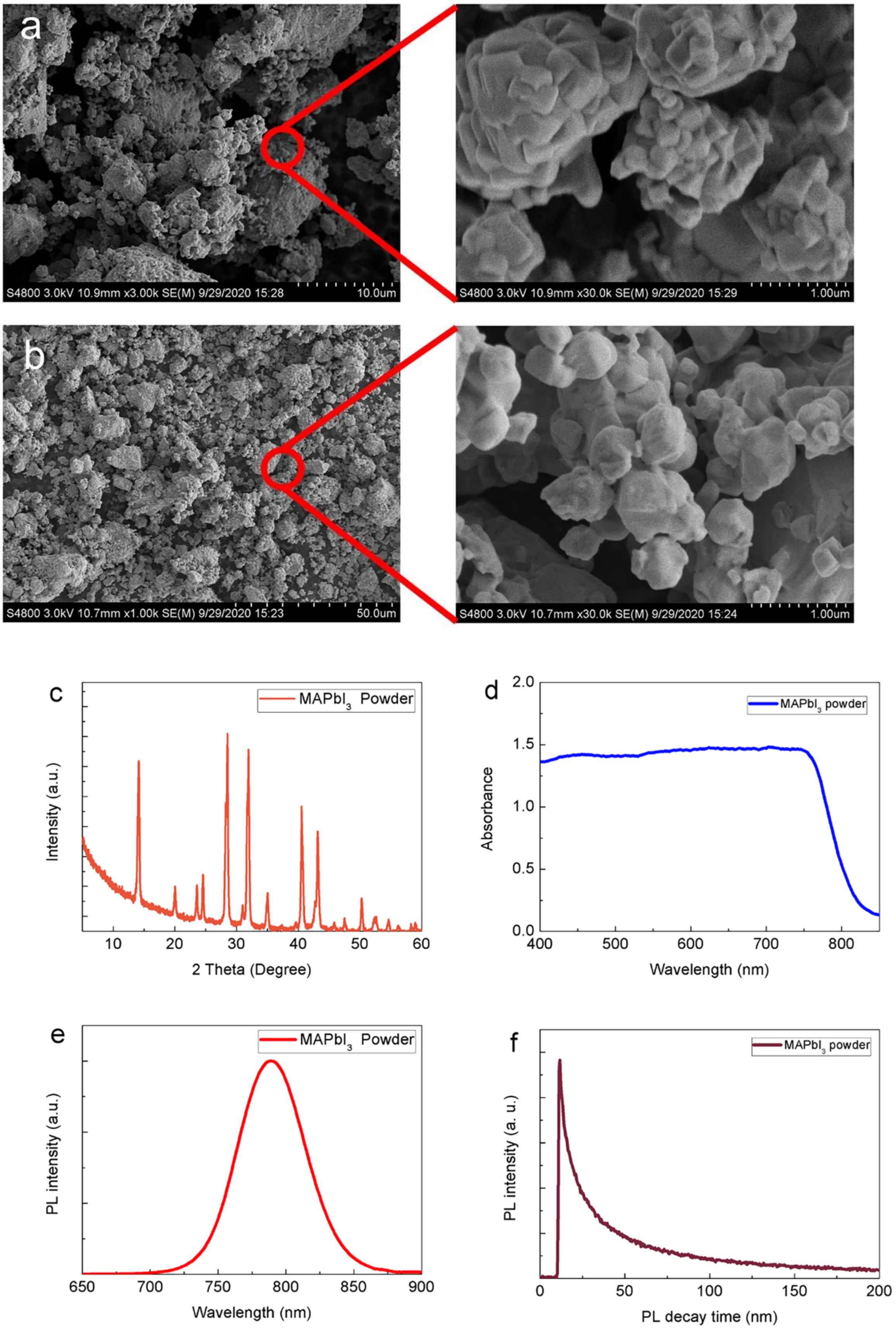

Figure S3. Characterisations of mechanosynthesised perovskite materials. a, SEM of $MAPbI_3$ perovskite powder. **b**, SEM of $MAPbI_3$ with 5 wt.% $PbCl_2$ perovskite powders. **c**, XRD

pattern; **d**, ultraviolet-visible–near-infrared absorption spectra; **e**, steady-state photoluminescence (PL) spectra and **f**, time-resolved PL decays of $MAPbI_3$ perovskite powder.

The characterization results (in **Figure S3**) of mechanosynthesised perovskite powder are consistent with the results of our previous report[3].

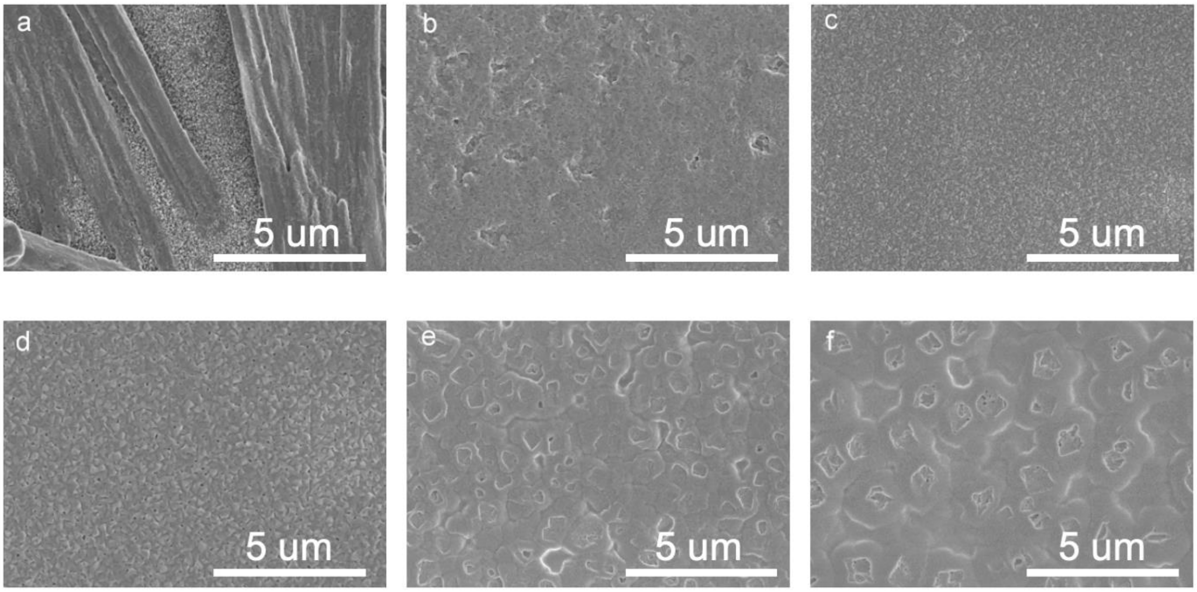

Figure S4. Top scanning electron microscopic images of **a**, $SMMAPbI_3$ film and **b**, $SMMAPbI_3$ film after MA gas treatment. **c**, $MSMAPbI_3$ film and **d**, $MSMAPbI_3$-A film. **e**, $MSMAPbI_3$-P film and **f**, $MSMAPbI_{3-x}Cl_x$ film after post-treatment. The same scale bar and label apply to all images in the column.

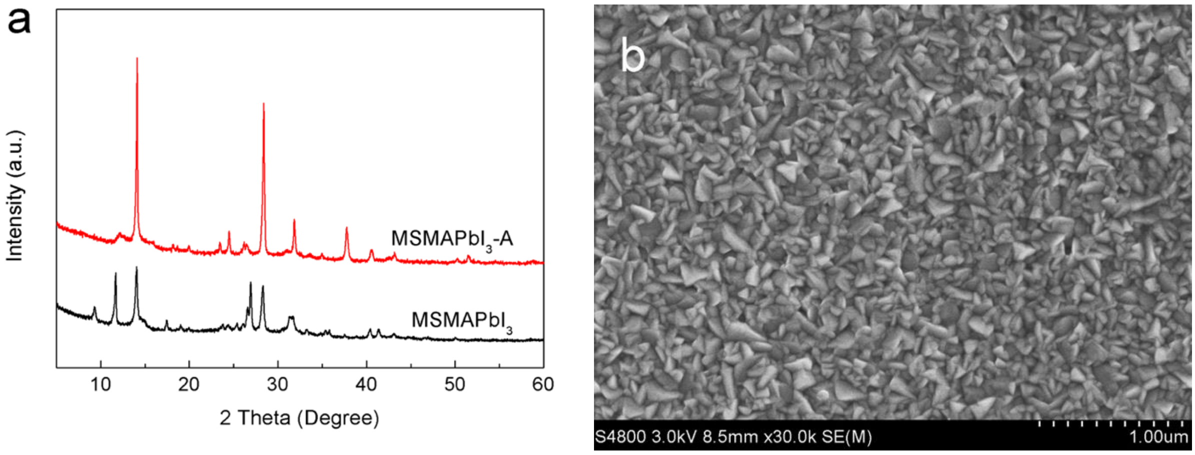

Figure S5. **a**, XRD patterns of perovskite films fabricated by magnetron sputtering ($MSMAPbI_3$) with annealing ($MSMAPbI_3$-A). **b**, SEM image of $MSMAPbI_3$.

As shown in **Figure S5a**, the XRD pattern of $MSMAPbI_3$ showed four peaks at 9.29 ^{o}, 11.67 ^{o}, 14.02 ^{o} and 28.26 ^{o} attributed to MAI, PbI_2 and $MAPbI_3$, respectively, revealing that magnetron sputtering technology caused the decomposition of a small part of perovskite in the process of film preparation. After annealing was performed through the heating stage ($MSMAPbI_3$-A), the peak of MAI disappeared, whilst that of PbI_2 significantly decreased, leading to the formation of perovskite from MAI and PbI_2. The excess PbI_2 was believed to be the sublimation of MAI in the process of annealing. The SEM image of $MSMAPbI_3$ is shown in **Figure S5b**. The $MSMAPbI_3$ film was made up of particles determined by the nature of the magnetron sputter technology. Therefore, post-treatment is essential for perovskite films prepared via magnetron sputtering.

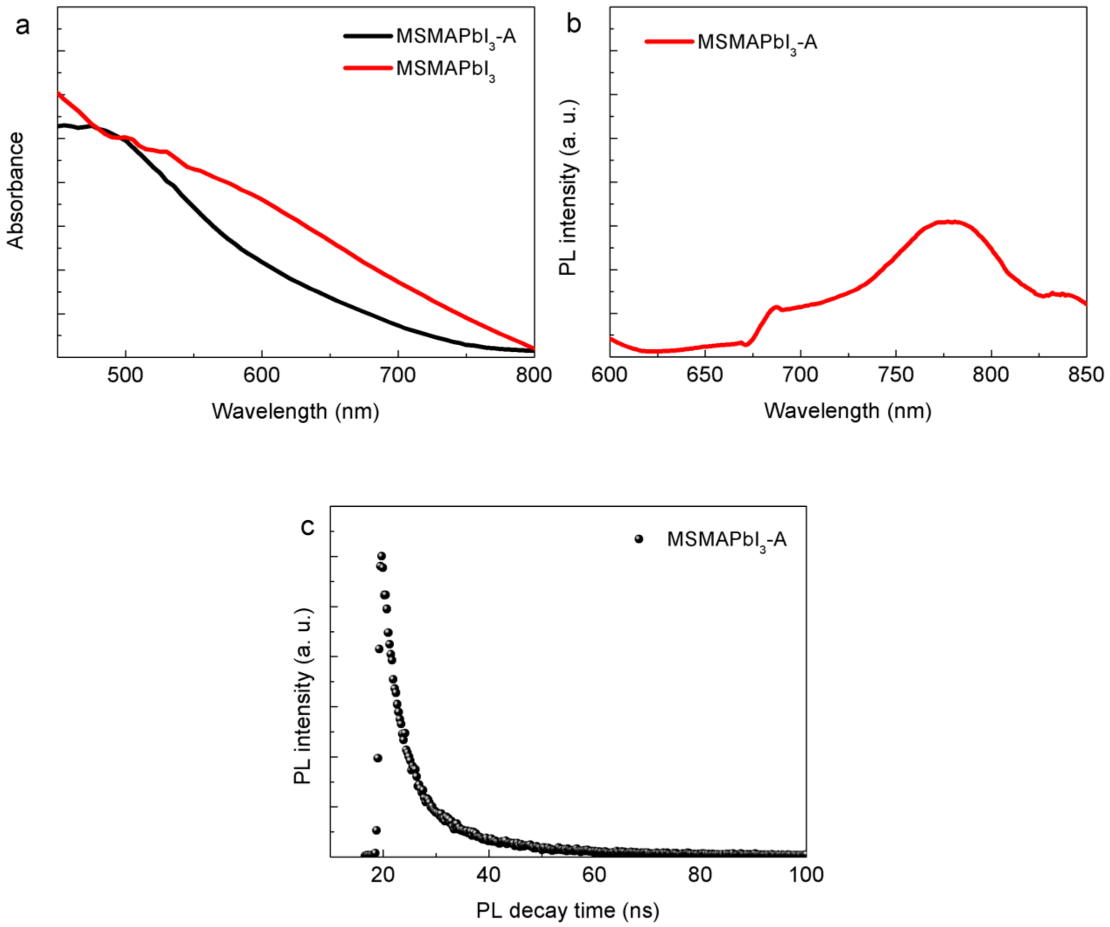

Figure S6. **a**, Ultraviolet-visible–near-infrared absorption spectra of $MSMAPbI_3$ and $MSMAPbI_3$-A films. **b**, Steady-state PL spectra of $MSMAPbI_3$-A. **c**, TRPL spectra of $MSMAPbI_3$-A

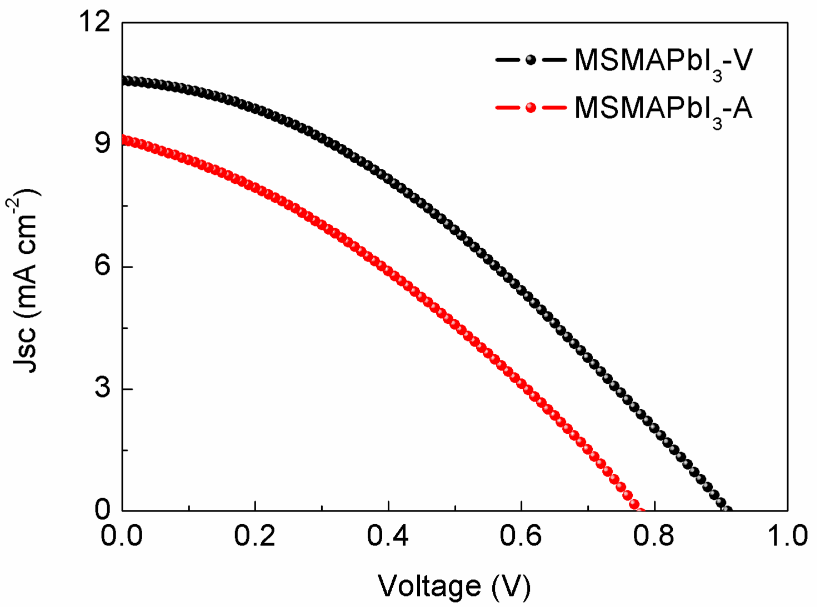

Figure S7. Device performance of $MSMAPbI_3$-V- and $MSMAPbI_3$-A-based solar cells.

The $MSMAPbI_3$-A-based device showed an open-circuit voltage (V_{OC}) of 0.78 V, a short-circuit current (J_{SC}) of 9.12 mA cm^{-2}, a fill factor (FF) of 0.33, and a power conversion efficiency (PCE) of 2.35 % under backward scan. The $MSMAPbI_3$-V-based device showed an open-circuit voltage (V_{OC}) of 0.91 V, a short-circuit current (J_{SC}) of 10.57 mA cm^{-2}, a fill factor (FF) of 0.36, and a power conversion efficiency (PCE) of 3.46 % under backward scan in **Figure S7** and **Table S2**.

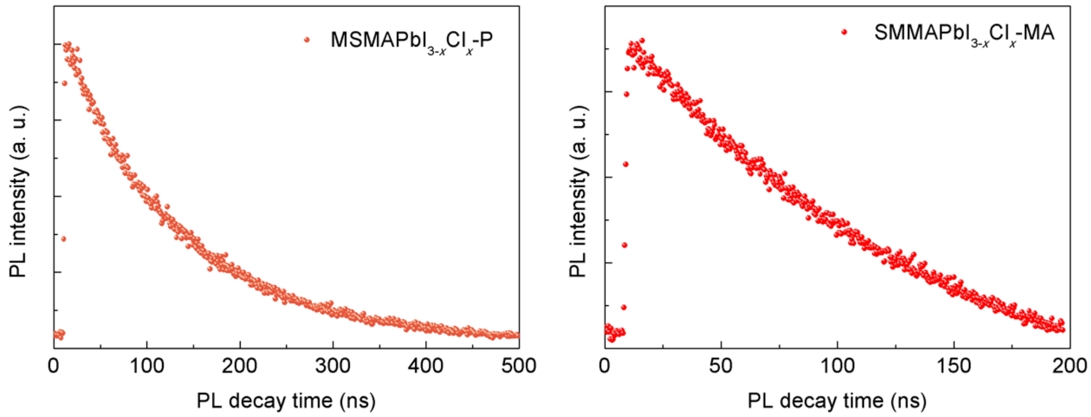

Figure S8. Time-resolved PL decays of $MSMAPbI_{3-x}Cl_x$-P and $SMMAPbI_{3-x}Cl_x$-MA films.

Time-resolved PL decay profiles were measured, in which balls indicate the measured data. The curves were fitted using a bi-exponential decay model ($y = A_1\exp[-(x–x_0)/ \tau_1] + A_2(\exp[-(x–x_0)/\tau_2])$.

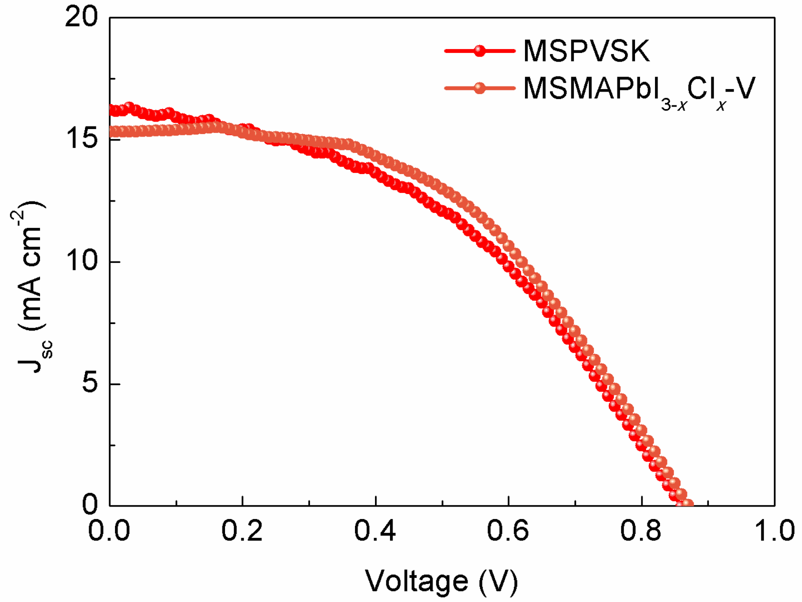

Figure S9. Device performance of MSMAPbI$_{3-x}$Cl$_x$-V- and MSMAPbI$_{3-x}$Cl$_x$-based solar cells.

MSPVSK films were prepared by magnetron sputtering without post-treatment of $MAPbI_3$ powders with a small amount of $MAPbCl_3$ powders. MSMAPbI$_{3-x}$Cl$_x$-V films were prepared by magnetron sputtering and vapor-assisted treatment with methanaminium iodide gas of $MAPbI_3$ powders with a small amount of $PbCl_2$ powders. The MSPVSK-based device showed an open-circuit voltage (V_{OC}) of 0.86 V, a short-circuit current (J_{SC}) of 16.22 mA cm^{-2}, a fill factor (FF) of 0.44, and a power conversion efficiency (PCE) of 6.14 % under backward scan. The MSMAPbI$_{3-x}$Cl$_x$-V-based device showed an open-circuit voltage (V_{OC}) of 0.87 V, a short-circuit current (J_{SC}) of 15.32 mA cm^{-2}, a fill factor (FF) of 0.5, and a power conversion efficiency (PCE) of 6.66 % under backward scan. After exploration and comparison, we chose the MSMAPbI$_{3-x}$Cl$_x$-V system as the research object and further optimize the conditions in this work.

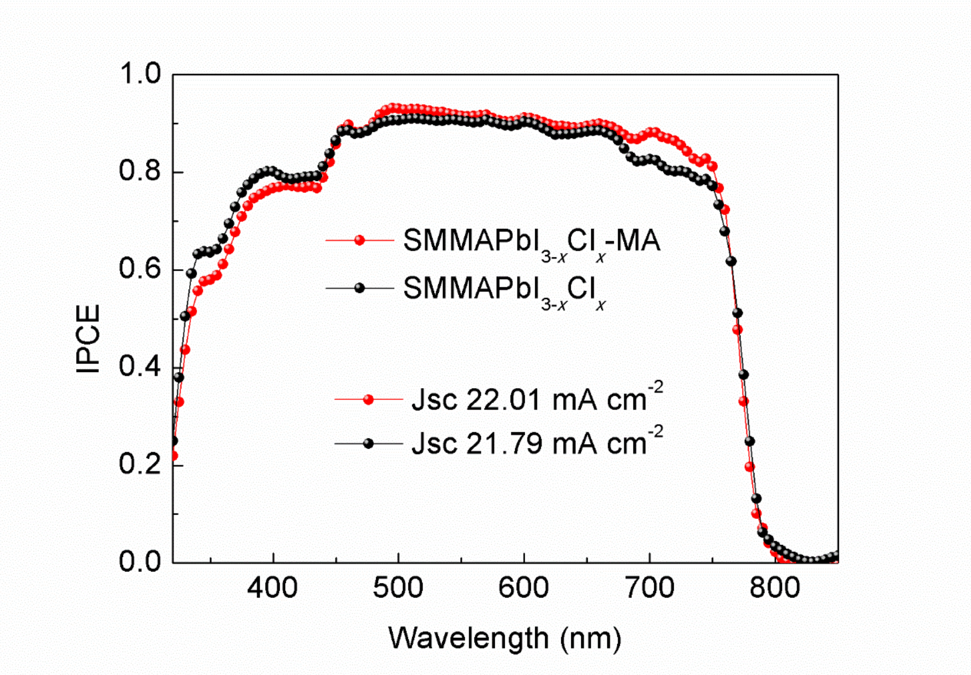

Figure S10. IPCE spectrum. The J_{SC} values of $SMMAPbI_{3-x}Cl_x$- and $SMMAPbI_{3-x}Cl_x$-MA-based devices calculated from the IPCE curve are 21.79 and 22.01 mA cm^{-2}, respectively.

Table S1 Film thickness as the sputtering time

Times (min)	2	4	6
Film thickness (nm)	210	390	580

The perovskite film thickness was measured as the time of sputtering methylammonium lead halide ($MAPbI_3$) prepared by mechanosynthesis on FTO/TiO_2 substrates, as shown in **Table S1**. When the sputter time was 2 min, the thickness of perovskite film was approximately 200 nm. As the sputtering time increased to 4 min, the perovskite film thickness increased to approximately 400 nm. After 6 min of sputtering, the thickness further increased to approximately 550 nm.

Table S2 Photovoltaic performance

	Jsc (mA cm^{-2})	Voc (V)	FF	PCE (%)
**$MSMAPbI_3$-A**	9.12	0.78	0.33	2.35
**$MSMAPbI_3$-V**	10.57	0.91	0.36	3.46
**$MSMAPbI_{3-x}Cl_x$-V**	15.32	0.87	0.5	6.66
**MSPVSK**	16.22	0.86	0.44	6.14

1. Q. Chen, H. Zhou, Z. Hong, S. Luo, H. S. Duan, H. H. Wang, Y. Liu, G. Li, Y. Yang, *J. Am. Chem. Soc.* **2013**, *136*, 622.
2. Z. Zhou, Z. Wang, Y. Zhou, S. Pang, D. Wang, H. Xu, Z. Liu, N. P. Padture, G. Cui, *Angew. Chem. Int. Ed.* **2015**, *54*, 9705.
3. S. Tang, X. Y. Xiao, J. Hu, B., Gao, H. L. Chen, Z. Y. Peng, J. C. Wen, M. Era, D. C. Zou, *Chemplusсhem* **2020**, *85*, 240.
4. H. Rao, S. Ye, W. Sun, W. Yan, Y. Li, H. Peng, Z. Liu, Z. Bian, Y. Li, C. A. Huang, *Nano Energy* **2016**, *27,* 51.
